# Primary Health Conditions Among Medical Crowdfunding Campaigns in the United States: Natural Language Processing Study

**DOI:** 10.2196/83413

**Published:** 2026-04-17

**Authors:** Shaojun Yu, Shu Liu, K Robin Yabroff, Farhad Islami, Fumiko Chino, Jing Zhang, Zhiyuan Zheng

**Affiliations:** 1 Surveillance, Prevention & Health Services Research Department American Cancer Society Atlanta, GA United States; 2 Computer Science Department Emory University Atlanta, GA United States; 3 Department of Breast Radiation Oncology MD Anderson Cancer Center Houston, TX United States

**Keywords:** medical crowdfunding, primary health conditions, natural language processing, crowdfunding economy, major health conditions, funds received and requested

## Introduction

Average US health care spending accounted for 20.4% of annual per capita income ($13,493 of $66,220; all monetary values are in US $) in 2022 [1]. Moreover, many Americans have limited liquid assets for unexpected out-of-pocket medical costs [2]. Therefore, peer-to-peer medical crowdfunding campaigns are increasingly used to raise money for medical expenses [3]. However, little evidence is available about which health conditions are driving increases in crowdfunding campaigns and the magnitude of unmet financial needs.

Manual review of crowdfunding campaigns is not feasible at scale. This study used a large natural language processing (NLP) model to empirically categorize US medical crowdfunding campaigns by primary health condition and to quantify funds requested, funds received, and resulting shortfalls between funds requested and received. The aim of this study was to identify the major health conditions driving medical crowdfunding and to characterize variation in fundraising outcomes across conditions.

## Methods

### Overview

A web scraper was manually developed in Python using standard HTML parsing techniques to systematically retrieve publicly available crowdfunding campaigns categorized as “medical” on the GoFundMe website and initiated between May 1, 2022, and May 31, 2023 [4]. Each campaign webpage includes a unique ID, the primary fundraising narrative, and donation records. Donation records were collected for 90 days, when most campaign activity occurs. The main fundraising stories typically describe the beneficiary’s health condition and specific needs. All campaigns were limited to those originating in the United States and written in English. All stories were preprocessed to remove special characters, emojis, and formatting artifacts before NLP classification.

**Figure 1:**
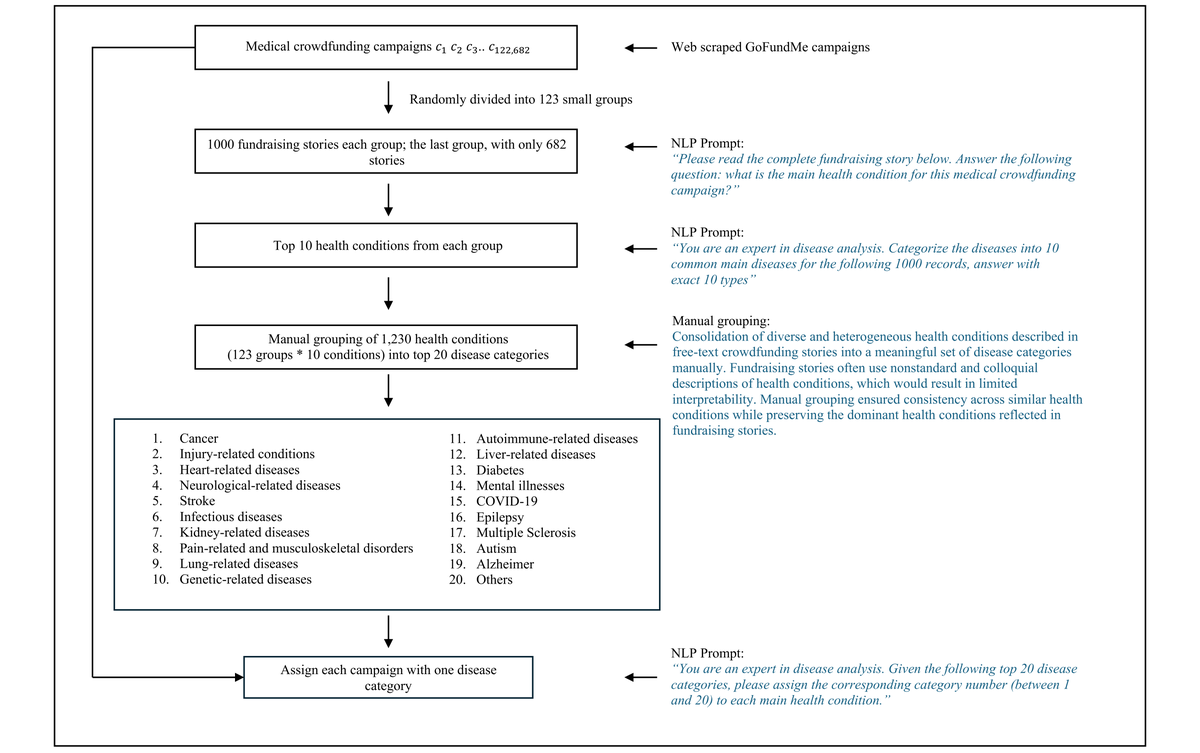
Diagram of the empirical approach used to extract primary health conditions from US medical crowdfunding campaigns.

An empirical multistage NLP approach was developed to categorize campaigns by health condition. A large language model (LLM; GPT-3.5 [OpenAI]; about 4000 words per query) was used to analyze fundraising stories, and for each campaign, the LLM was instructed to identify a single primary health condition most directly related to the fundraising purpose (Figure 1). As GPT-3.5 is most efficient in grouping tasks when analyzing 500 to 1500 words, we sequentially divided all campaigns into smaller groups, with 1000 in each group. The same NLP model was used to obtain the top 10 most mentioned health conditions from each small group. Because fundraising stories use diverse and nonstandard descriptions of health conditions, which are typically not consistent with standardized taxonomies such as the *International Classification of Diseases, 10th Revision* (*ICD-10*), we manually consolidated related terms into broader disease categories to improve interpretability and consistency. The most common 20 categories (19 conditions plus “others”; keywords for each condition are shown in the table below) were selected based on the number of times that condition appeared on the list of 1230 health conditions. We followed the Strengthening the Reporting of Observational Studies in Epidemiology (STROBE) reporting guideline for all summary statistics [5].

### Ethical Considerations

This study was deemed exempt from ethical approval by the Morehouse School of Medicine Institutional Review Board.

## Results

In total, 122,682 medical crowdfunding campaigns were identified (Table 1). Cancer was the leading health condition by volume (n=51,843, 42.3% of all campaigns), followed by injury-related conditions (n=10,423, 8.5%), heart-related disease (n=8659, 7.1%), and neurological disease (n=6248, 5.1%).

**Table 1:** Funds requested and received by health condition among medical crowdfunding campaigns in the United States. All medical crowdfunding campaigns were retrieved from the publicly available GoFundMe website that were initiated between May 1, 2022, and May 31, 2023, and followed up with 90 days of donation records.

Health conditions	Crowdfunding campaigns, n (%)	Campaigns with funds requested and received, n^a^	Total amount of funds requested^b^ vs received^c^ (US $), n/N (%)	Funds received per campaign (US $), mean (SD)	Funds requested per campaign (US $), mean (SD)	Median funds requested per campaign (US $), median, (IQR)	Median funds received per campaign (US $), median, (IQR)	All campaigns reaching fundraising goals (%)^b,c^	Campaigns reaching fundraising goals that received >US $50,000) (%)^b,c^
All health conditions	122,682 (100.0)	112,198	1,871,526,583/575,914,886 (30.8)	5133 (8880)	16,681 (22,692)	10,000 (5000-20,000)	2207 (860-5730)	10,214 (9.2)	755 (0.68)
Cancer	51,843 (42.3)	48,871	889,109,288/273,959,992 (30.8)	5606 (8044)	18,192 (21,660)	10,000 (5000-20,000)	2880 (1130-6990)	4,072 (8.4)	260 (0.53)
Injury-related conditions^d^	10,423 (8.5)	9564	191,429,273/59,966,728 (31.3)	6270 (13,407)	20,015 (32,019)	10,000 (5000-20,000)	2060 (810-5722)	939 (9.8)	186 (1.94)
Heart-related diseases	8659 (7.1)	8027	123,384,193/37,895,760 (30.7)	4721 (8512)	15,371 (20,836)	10,000 (5000-18,000)	1954 (795-5004)	827 (10.3)	51 (0.64)
Neurological diseases^e^	6248 (5.1)	5380	97,947,309/32,332,547 (33.0)	6010 (11,856)	18,206 (26,590)	10,000 (5000-20,000)	2186 (835-6012)	634 (11.8)	74 (1.38)
Stroke	5360 (4.4)	4902	93,430,952/29,217,036 (31.3)	5960 (10,883)	19,060 (26,170)	10,000 (5000-20,000)	2462 (970-6434)	467 (9.5)	56 (1.14)
Infectious diseases	4540 (3.7)	4149	54,009,867/17,758,936 (32.8)	4280 (7640)	13,018 (17,880)	6607 (3500-15,000)	1783 (710-4525)	437 (10.5)	28 (0.67)
Kidney-related diseases	4027 (3.3)	3698	55,918,536/14,109,460 (25.2)	3815 (7213)	15,121 (21,880)	8000 (4000-18,000)	1600 (655-3950)	329 (8.9)	18 (0.49)
Pain-related and musculoskeletal disorders^f^	3950 (3.2)	3429	43,462,058/11,993,688 (27.6)	3498 (6588)	12,675 (17,387)	7000 (3500-15,000)	1440 (615-3700)	376 (11.0)	12 (0.35)
Lung-related diseases^g^	3090 (2.5)	2855	37,071,628 /12,351,260 (33.3)	4326 (7767)	12,985 (17,943)	6000 (3500-15,000)	1790 (767-4432)	358 (12.5)	16 (0.56)
Genetic-related diseases^h^	2868 (2.3)	2428	42,152,956/12,173,939 (28.8)	5014 (8650)	17,361 (28,849)	9032 (4000-20,000)	2080 (865-5171)	310 (12.8)	15 (0.62)
Autoimmune-related diseases^i^	2379 (1.9)	2140	29,812,924/8,870,851 (29.8)	4145 (6791)	13,931 (18,840)	7500 (4000-15,000)	1877 (750-4503)	241 (11.3)	5 (0.23)
Liver-related diseases	2079 (1.7)	1934	36,243,923/8,888,493 (24.5)	4596 (7647)	18,740 (30,296)	10,000 (5000-20,000)	2009 (840-5024)	173 (8.9)	7 (0.36)
Diabetes	1971 (1.6)	1840	22,875,850/5,527,402 (24.2)	3004 (5471)	12,433 (17,823)	7000 (3500-14,000)	1290 (590-3123)	143 (7.8)	4 (0.21)
Mental illnesses^j^	1543 (1.3)	1263	14,448,719/4,573,398 (31.6)	3621 (6387)	11,440 (14,259)	6000 (3000-14,000)	1420 (555-3877)	142 (11.3)	4 (0.32)
COVID-19	1118 (0.9)	1007	13,909,965/4,547,831 (32.7)	4516 (8591)	13,813 (18,508)	7500 (3463-15,000)	1815 (756-4305)	117 (11.6)	6 (0.60)
Epilepsy	977 (0.8)	836	10,571,567/3,201,893 (30.3)	3830 (6256)	12,645 (16,904)	7000 (3000-15,000)	1670 (664-4501)	104 (12.5)	3 (0.36)
Multiple sclerosis	806 (0.7)	659	9,947,287/3,170,741 (31.8)	4811 (8818)	15,095 (18,540)	10,000 (4000-16,000)	1920 (840-5205)	74 (11.3)	4 (0.61)
Autism	552 (0.4)	440	4,937,909/1,491,344 (30.2)	3389 (6528)	11,223 (14,275)	6000 (3000-15,000)	1407 (570-3096)	50 (11.4)	2 (0.45)
Alzheimer disease	501 (0.4)	386	7,087,213/2,388,499 (33.7)	6188 (13,656)	18,361 (25,238)	10,000 (5000-20,000)	1972 (827-5327)	41 (10.7)	6 (1.55)
Others	9748 (7.9)	8390	1,871,526,583/575,914,886 (33.5)	3754 (7,390)	11,177 (15,410)	5200 (3000-10,000)	1455 (580-3864)	1,059 (12.7)	43 (0.51)

^a^Crowdfunding campaigns without fundraising goals or without any donations were excluded.

^b^The calculation of funds requested (ie, fundraising goals) excluded missing values and those <1st percentile and >99th percentile.

^c^For campaigns for which the funds received reached or exceeded fundraising goals, percentages were capped at 100% (ie, all those >1 were set as 100%).

^d^Injury-related conditions included the following terms: *accidents*, *injuries*, *trauma*, *fractures*, and *amputations*.

^e^Neurological-related diseases included the following terms: *neurological disorders*, *neurological conditions*, and *cerebral palsy*.

^f^Pain-related and musculoskeletal disorders included the following terms: pain, chronic pain, chronic pain syndromes, osteoarthritis, musculoskeletal, and muscular dystrophy.

^g^Lung-related diseases included the following terms: *respiratory diseases*, *respiratory issues*, *lung diseases*, *COPD*, *pneumonia*, and *pulmonary fibrosis*.

^h^Genetic-related diseases included the following terms: *genetic diseases*, *genetic disorders*, *Ehlers-Danlos syndrome*, *Down syndrome*, and *Crouzon syndrome*.

^i^Autoimmune-related diseases included the following terms: *autoimmune diseases*, *autoimmune disorders*, *lupus*, and *rheumatoid*.

^j^Mental illnesses included the following terms: *mental illnesses* and *mental health*.

The majority (n=112,198, 91.5%) of campaigns listed fundraising goals and received donations; among which, the total amount received ($575,914,886) accounted for 30.8% of the total funds requested ($1,871,526,583). Only 9.2% achieved their fundraising goals within 90 days after campaign initiation.

Cancer-related campaigns received the highest amounts of financial assistance (median $2880, IQR $1130-$6990 per campaign), followed by stroke ($2462, IQR $970-$6434), neurological diseases ($2186, IQR $835-$6012), and injury-related conditions ($2060, IQR $810-$5772), whereas diabetes received the lowest amounts (median: $1290, IQR $590-$3123). Alzheimer’s disease had the highest proportion of funds received relative to funds requested (33.7%), driven by a relatively higher share of campaigns that raised ≥$50,000 (1.55%).

Manual review of 500 randomly selected crowdfunding stories showed condition classification accuracy of 92.3%, consistent with previous literature [3].

## Discussion

In this contemporary analysis of more than 100,000 US medical crowdfunding campaigns over a 13-month period, we identified the 20 most common categories, which collectively raised $575,914,886 during the 90-day follow-up. The frequency at which health conditions were mentioned in crowdfunding campaigns does not reflect their prevalence at the population level, but instead aspects of financial hardship due to the condition [6]. As such, the total funds requested and the proportion actually received varied greatly by health condition, with the median cancer crowdfunding campaign raising more than twice as much as that for diabetes.

Cancer-related crowdfunding campaigns also represent nearly the combined number of campaigns of all other 18 common health conditions. Moreover, cancer-related campaigns received the most donations but, due to higher fundraising goals, these campaigns raised proportionately less of the requested total [7]. Previous research showed that cancer-related crowdfunding campaigns are often initiated due to high out-of-pocket medical costs and unmet social needs (ie, food, housing, and transportation), as patients experience disruptions in employment, insurance, and income following diagnosis [3,8]. Moreover, campaign success depends on social networks, visibility, and donors’ capacity to give—factors that vary by socioeconomic status and geography and may potentially widen disparities in access to cancer care [9].

A limitation of this study is that health conditions identified through NLP analyses cannot be verified by medical records. Although standardized taxonomies such as *ICD-10* provide well-established disease classifications, they are rarely mentioned in fundraising stories written by patients and informal caregivers. Our empirical approach focused on how health conditions were described in crowdfunding stories, which may not map cleanly to *ICD-10* codes nor capture multiple conditions; future work could explore systematic mappings to improve comparability. At the time this study was conducted, GPT-3.5 was the available LLM and was therefore used; subsequent advances in LLM availability may allow future studies to conduct similar large-scale analyses using different LLMs. Because this study examined only GoFundMe campaigns, the findings may reflect platform-specific dynamics and may not be generalizable to other crowdfunding platforms. The proportion of funds received relative to requested amounts reflects relative unmet need, whereas total requested amounts represent absolute financial burden. Accordingly, low goal attainment should not be interpreted as inefficiency or failure of crowdfunding. The observed shortfalls underscore persistent financial challenges faced by patients and families and highlight the need for further research and policy attention.

## Data Availability

The data analyzed in this manuscript are publicly available from the GoFundMe website [4].

## Abbreviations

ICD-10: International Classification of Diseases, 10th Revision
LLM: large language model
NLP: natural language processing
STROBE: Strengthening the Reporting of Observational Studies in Epidemiology
